# An iRGD Based Strategy to Study Electrochemically the Species Inside a Cell

**DOI:** 10.3390/ijms130810424

**Published:** 2012-08-21

**Authors:** Limin Ning, Xiaoxi Li, Xiaorong Ding, Yongmei Yin, Genxi Li

**Affiliations:** 1 Department of Biochemistry and State Key Laboratory of Pharmaceutical Biotechnology, Nanjing University, Nanjing 210093, China; E-Mails: ninglimin2006@126.com (L.N.); lxx.zlxhaoyou@yahoo.com.cn (X.L.); 2 Laboratory of Biosensing Technology, School of Life Sciences, Shanghai University, Shanghai 200444, China; E-Mail: dingxiaoronglaofuzi@126.com; 3 Department of Oncology, the First Affiliated Hospital of Nanjing Medical University, Nanjing 210029, China; E-Mail:ym.yin@hotmail.com

**Keywords:** iRGD, cell penetration, doxorubicin, cell adhesion, electrochemistry

## Abstract

This paper reports a method for electrical communication between the inner part of cells and an electrode with the help of iRGD peptide. Due to the enhancement of the cell penetration caused by iRGD peptide, DNA molecules, previously modified on a gold electrode surface, can be easily transfected into the cells. At the same time, doxorubicin, an anticancer drug, can also be transfected into cells with high penetration. Consequently, doxorubicin binds to DNA chains through electrostatic interaction, and the redox reaction is transferred out of the cell across the cell membrane. As a result, this work may provide a novel way to get information from inside of cells.

## 1. Introduction

Electrochemical techniques have been employed for the study of not only small molecules, but also biological macromolecules such as proteins [1,2] and nucleic acids [3,4]. With the result that researchers are trying to probe into cellular systems. The detection of cells in quantity [5], the morphology [6] and the electroanalysis of cell apoptosis [7–9] have all been conducted. Nevertheless, due to the nonconductive cell membrane, electrochemical signals inside cells cannot be easily obtained [10]. In this work we have taken advantage of the effect of iRGD (CRGDK/RGPD/EC) on cell penetration and proposed another way for electrochemical communication between an electrode and the species inside cells.

iRGD is a kind of tumor-penetrating peptide which can enhance the permeability of tumor cells mediated by integrins and neuropilin-1 (NRP-1) upregulated on the cells [11,12]. In the process of enhancing the penetration of tumor cells, the RGD motif mediates binding to αν integrins firstly on tumor endothelium followed by a proteolytic cleavage, exposing a binding motif for NRP-1, which can mediate penetration into tissue and cells. So, when the peptide is co-administered with doxorubicin, which exhibits a wide spectrum of activity against solid tumors, lymphomas, and leukemias [13], the enhanced cell penetration caused by iRGD allows more doxorubicin influx into the cells, while DNA molecules are taken up into the cells. Since one end of the DNA molecule has been immobilized on a gold electrode surface, with doxorubicin binding to the DNA chains, the redox reaction of doxorubicin inside the cells as an electroactive substance whose redox centers are quinone and hydroquinone groups [14] can be obtained and read out by way of electron transfer through DNA chains. Therefore, the electrical communication between the inner part of the cell and the electrode is made available and a way to electrochemically study the species inside the cells is presented.

## 2. Results and Discussion

**Scheme 1:**
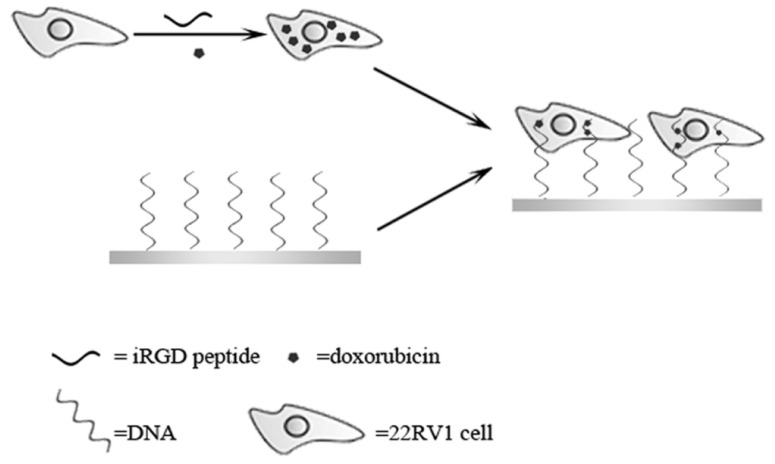
Schematic illustration of the electrical communication between the inner part of cells and an electrode.

The strategy for electrical communication between the inner part of cells and the electrode is schematically depicted in Scheme 1. Firstly, DNA molecules rich in CpG sequences are immobilized on a gold electrode. Then the electrode is immersed in the cell suspension which has been pretreated with iRGD and doxorubicin. In this process, the enhanced cell penetration by iRGD will promote the uptake of not only doxorubicin but also DNA molecules, one end of which has been modified on the electrode, in the cells. Since doxorubicin inside the cells will bind to DNA chains by electrostatic interaction, electron transfer from the redox species inside the cells across the cell membrane to the electrode can be achieved.

Since the cells are loaded with the anticancer drug doxorubicin, exposed to cell penetrating peptides and penetrated with DNA, this series of treatments will ultimately affect the cell membrane and its electrical potential and could trigger apoptosis. Therefore, cytotoxicity studies were carried out to ensure that the signal comes from the drugs inside the cells and that cell apoptosis and death has not occurred after the above pretreatment. In this study, *f* test and *t* test were used to determine the effects of doxorubicin and iRGD on cell growth. Since no significant differences (*p* > 0.05) can be observed between the growth in the presence and absence of doxorubicin and/or iRGD, cell apoptosis and death did not occur before the electrochemical experiments were performed.

**Figure 1:**
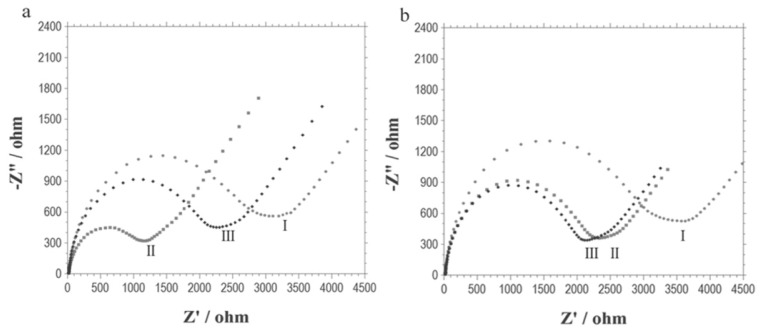
Electrochemical impedance spectra (Nyquist plots) of the DNA modified electrode (I) before and (II) after its incubation with (**a**) 22Rv1 cells which were treated with both 100 μM iRGD and 50 μM doxorubicin, or (**b**) 50 μM doxorubicin. Curve (III) is the case of further treatment with cell lysis buffer; Test solution: 1 M KNO_3_ containing 5 mM Fe(CN)_6_^3−/4−^. Biasing potential: 0.222 V. Amplitude: 5 mV. Frequency range: 0.1 Hz to 100 kHz.

Electrochemical impedance spectroscopy (EIS) has been employed to characterize modification of the electrode in this work. It can be observed from Figure 1a that the interfacial electron transfer resistance of the DNA-modified electrode is greatly decreased after incubation of the electrode with 22Rv1 cells in the presence of iRGD, because the cells have been assembled on the electrode surface. The explanation is very clear, since DNA molecules are negatively charged, they repel the electroactive probes [Fe(CN)_6_]^3−/4−^. So, the interfacial electron transfer resistance of the DNA-modified electrode is very high. Nevertheless, after the DNA molecules are transfected into the cells, the negative charges are shielded, thus the weaker repellence leads to decrease in the interfacial electron transfer resistance. Figure 1a also shows that the resistance will jump back if the electrode is further treated with cell lysis buffer overnight, due to breaking down of the shield of negative charge, although the resistance cannot return to the same level as that of the original DNA-modified electrode. This is because the binding of the positively charged doxorubicin to DNA has partially neutralized the negatively charged DNA molecules. The explanation is confirmed by Figure 1b, which shows that the interfacial electron transfer resistance of the DNA-modified electrode is decreased significantly after the incubation of the electrode with doxorubicin; while further treatment with cell lysis has little effect on the resistance.

**Figure 2:**
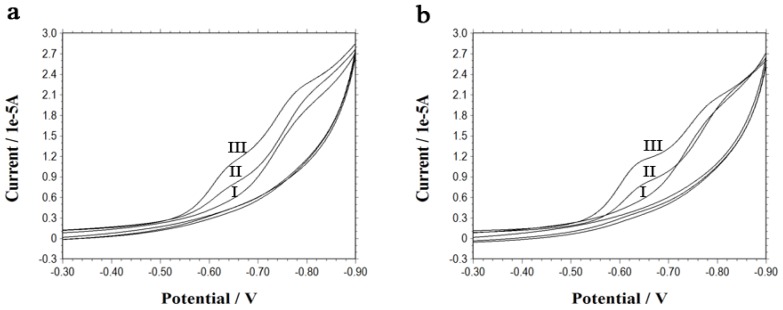
Cyclic voltammograms for a 50 mM Tris-HCl buffer (pH 7.4) obtained at the DNA-modified electrode after its incubation with 22Rv1 cells which were previously pretreated by (**a**) 50 μM doxorubicin and (I) 0 μM, (II) 20 μM, (III) 100 μM iRGD, or (**b**) 100 μM iRGD and (I) 0 μM, (II) 50 μM, (III) 100 μM doxorubicin. Scan rate: 100 mV s^−1^.

Cyclic voltammetry (CV) was employed to investigate the electrical communication between the gold electrode and the electroactive species inside the cells. Figure 2a presents a reduction peak appearing at −0.64 V which is attributable to the reduction of quinone in doxorubicin [15,16]. The peak appears only after the incubation of the electrode with the cell suspension pretreated by iRGD, and the peak current is directly proportional to the concentration of iRGD if the concentration of doxorubicin remains unchanged. The results reveal that iRGD peptide has indeed enhanced the cell penetration, thus the enhancement of the cell penetration caused by iRGD improves the uptake of both DNA molecules modified on the electrode and doxorubicin, the electroactive drug molecule. With more iRGD acting on cells, more doxorubicin molecules enter into the cells and transfer their redox reactions out through DNA chains. Thus a higher electrochemical signal is obtained.

The relationship between the doxorubicin concentration inside the cells and the obtained peak current was also examined in this work. Experimental results show that the peak current increases with the concentration of doxorubicin (Figure 2b). In this case, the enhanced cell penetration is kept unchanged, since the concentration of the iRGD peptide is set at 100 μM, leading to a constant uptake of DNA molecules. Nevertheless, with the increase of the concentration of doxorubicin added to the cell suspension, a steadily enhanced influx of doxorubicin inside the cells is achieved, thus a higher redox peak current can be observed.

**Figure 3:**
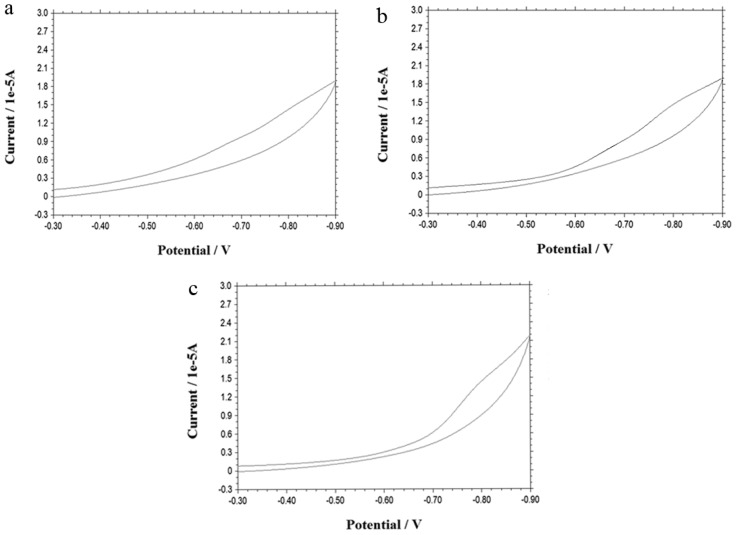
(**a**) Cyclic voltammogram for a 50 mM Tris-HCl buffer (pH 7.4) obtained at the bare gold electrode after its incubation with 22Rv1 cells which were previously pretreated by 50 μM doxorubicin and 100 μM iRGD; (**b**) Cyclic voltammogram for a 50 mM Tris-HCl buffer (pH 7.4) obtained at the DNA-modified electrode after its incubation with 22Rv1 cells which were previously pretreated by 50 μM doxorubicin and 100 μM control peptide with its sequence LRRASLGGGGC; (**c**) Cyclic voltammogram for a 50 mM Tris-HCl buffer (pH 7.4) obtained at the DNA-modified electrode after its incubation with 293T cells which were previously pretreated by 50 μM doxorubicin and 100 μM iRGD.

It should be mentioned that without DNA being modified on the electrode surface, no peak of doxorubicin can be obtained (Figure 3a), since no molecules are taken up into the cells, thus the electric communication between the inner part of the cells and the electrode cannot be achieved. On the other hand, other peptides do not work either. As is shown in Figure 3b, when the control peptide instead of iRGD is employed, no peak can be observed. Futhermore, when control cells, for instance, 293T cells, were employed for this study, no peak could be observed either.

## 3. Experimental Section

### 3.1. Chemicals and Reagents

iRGD peptide (CRGDKGPDC) and control peptide (LRRASLGGGGC) were synthesized by China Peptides Co., Ltd. The thiolated DNA, 5′-HS-(CH_2_)_6_-CAC GAC GTT GTA AAA CGA CGG CCA GAG CAG-3′, was obtained from Shanghai Invitrogen Biotechnology Co., Ltd. Trypsin, mercaptohexanol (MCH), tris(2-carboxyethyl)phosphine hydrochloride (TCEP), ethylenediaminetetraacetic acid (EDTA) and doxorubicin (hydrochloride form; 98%) were purchased from Sigma. Fetal bovine serum and dulbecco’s modified Eagle medium (DMEM) were obtained from Nanjing Sunshine Biotechnology Co., Ltd. The Cell Counting Kit-8 (CCK-8) was obtained from Beyotime Institute of Biotechnology. The cell lines, human 22Rv1 prostate cancer cell and 293T cell were provided by Institute of Biochemistry and Cell Biology, Chinese Academy of Science. All the other chemicals were of analytical grade and used as received. All solutions were prepared with doubly distilled water, which was purified with a Milli-Q purification system (Barnstead) to a specific resistance of >18 MΩ cm.

### 3.2. Preparation of the DNA Modified Electrode

The thiolated DNA could be immobilized onto a gold electrode (3 mm diameter) via gold-sulfur chemistry. Before the surface modification, the electrode was first soaked in piranha solution (98% H_2_SO_4_:30% H_2_O_2_ = 3:1) for 5 min (Caution: Piranha solution reacts dangerously with organic matter!). Then, it was polished carefully on P3000 silicon carbide paper and alumina slurry (1 μm, 0.3 μm, 0.05 μm), respectively. After that, it was thoroughly washed by ultrasonicating in both ethanol and doubly distilled water for about 5 min. After the above pretreatment, the electrode was electrochemically cleaned to remove any remaining impurities. After drying with nitrogen, the gold electrode was immersed in the 10 mM Tris-HCl buffer solution containing 1 μM thiolated DNA, 1 mM EDTA, 1.0 M NaCl, and 1 mM TCEP (pH 8.0), for 16 h, followed by 1 h treatment with MCH. Finally, the electrode was thoroughly rinsed with pure water and dried again with nitrogen, and was ready for further experimentation.

### 3.3. Cell Culture

The human 22Rv1 prostate cancer cells overexpressing αν integrins and NRP-1 were cultured in DMEM supplemented with 10% (v/v) fetal bovine serum at 37 °C in a water-saturated incubator with 5% CO_2_. Before the treatment of iRGD, the cells were washed with phosphate buffered saline (PBS) twice and detached by Trypsin-EDTA for 1~3 min. The cells were then harvested by centrifugation (1000 rpm, 5 min) and resuspended in the same culture medium with the concentration of 1 × 10^6^ cell/mL. After that, iRGD peptide and doxorubicin dissolved in PBS with a series of concentrations were added to the cell suspension prepared above and incubated at 37 °C for 2 h. The cell suspension was then centrifugated, and re-suspended to remove the redundant iRGD and doxorubicin which remained outside the cells. Subsequently, the above prepared DNA-modified gold electrode was immersed in the cell solution and incubated at 37 °C for 6 h to achieve adhesion of cells onto the surface of the electrode.

### 3.4. Cytotoxicity Assays

To study cytotoxicity, suspension cell lines were plated in 96-well tissue culture plates at a density of 5000 cells per well. After 16 h, cells were attached. Doxorubicin, or the drug together with iRGD peptide were then added in the culture medium. Doxorubicin was studied with concentrations of 0 μM, 50 μM and 100 μM, and iRGD peptide at 0 μM, 20 μM and 100 μM. The final volume of medium was 100 μL. After incubating for 2 h, the culture medium was removed and fresh culture medium was added. After incubating for an additional 6 h, 10 μL CCK-8 solution was added to each well and the culture plates were returned to the incubator for 1 h. Finally, the absorbance at 450 nm was read for each well using a Micro plate spectrophotometer.

### 3.5. Electrochemical Measurements

Electrochemical impedance spectroscopy (EIS) and cyclic voltammetry (CV) were performed on a model 660C electrochemical analyzer (CH Instruments) at room temperature around 25 °C. For all the electrochemical measurements, a three-electrode system consisting of the modified gold electrode as the working electrode, a saturated calomel reference electrode (SCE) and a platinum auxiliary electrode was used. EIS was carried out with 1 M KNO_3_ containing 5 mM Fe(CN)_6_^3−/4−^ in the frequency range of 0.1 Hz to 100 kHz. The biasing potential was 0.222 V and amplitude was 5 mV. The CV experiment was performed with 50 mM Tris-HCl buffer (pH 7.0) with a scan rate of 100 mV s^−1^.

## 4. Conclusions

To summarize, this work developed an approach to look inside cells with the help of tumor-penetrating peptide, iRGD. Compared with other transfection reagents such as Ca^2+^ [10], the iRGD peptide, which also exists in fibronectin, performs cell penetration more gently and maintains cell viability at its best. This method may also have good selectivity towards tumor cells, since the iRGD peptide contains the RGD motif and the C-end rule peptide. As a consequence, this work could open more opportunities in the future for electrochemical studies of species inside cells.

## References

[1] Lebedev N., Trammell S.A., Tsoi S., Spano A., Kim J.H., Xu J., Twigg M.E., Schnur J.M. (2008). Increasing efficiency of photoelectronic conversion by encapsulation of photosynthetic reaction center proteins in arrayed carbon nanotube electrode. Langmuir.

[2] Peng Y., Jiang D., Su L., Zhang L., Yan M., Du J., Lu Y., Liu Y.N., Zhou F. (2009). Mixed monolayers of ferrocenylalkanethiol and encapsulated horseradish peroxidase for sensitive and durable electrochemical detection of hydrogen peroxide. Anal. Chem.

[3] Drummond T.G., Hill M.G., Barton J.K. (2003). Electrochemical DNA sensors. Nat. Biotechnol.

[4] Zhang J., Song S., Wang L., Pan D., Fan C. (2007). A gold nanoparticle-based chronocoulometric DNA sensor for amplified detection of DNA. Nat. Protoc.

[5] Matsumoto A., Sato N., Kataoka K., Miyahara Y. (2009). Noninvasive sialic acid detection at cell membrane by using phenylboronic acid modified self-assembled monolayer gold electrode. J. Am. Chem. Soc.

[6] Wischerhoff E., Uhlig K., Lankenau A., Borner H.G., Laschewsky A., Duschl C., Lutz J.F. (2008). Controlled cell adhesion on PEG-based switchable surfaces. Angew. Chem. Int. Ed. Engl.

[7] Du D., Cai J., Ju H., Yan F., Chen J., Jiang X., Chen H. (2005). Construction of a biomimetic zwitterionic interface for monitoring cell proliferation and apoptosis. Langmuir.

[8] Liu T., Zhu W., Yang X., Chen L., Yang R., Hua Z., Li G. (2009). Detection of apoptosis based on the interaction between annexin V and phosphatidylserine. Anal. Chem.

[9] Xiao H., Liu L., Meng F., Huang J., Li G. (2008). Electrochemical approach to detect apoptosis. Anal. Chem.

[10] Meng F., Yang J., Liu T., Zhu X., Li G. (2009). Electric communication between the inner part of a cell and an electrode: the way to look inside a cell. Anal. Chem.

[11] Sugahara K.N., Teesalu T., Karmali P.P., Kotamraju V.R., Agemy L., Girard O.M., Hanahan D., Mattrey R.F., Ruoslahti E. (2009). Tissue-penetrating delivery of compounds and nanoparticles into tumors. Cancer Cell.

[12] Sugahara K.N., Teesalu T., Karmali P.P., Kotamraju V.R., Agemy L., Greenwald D.R., Ruoslahti E. (2010). Coadministration of a tumor-penetrating peptide enhances the efficacy of cancer drugs. Science.

[13] Swift L.P., Rephaeli A., Nudelman A., Phillips D.R., Cutts S.M. (2006). Doxorubicin-DNA adducts induce a non-topoisomerase II-mediated form of cell death. Cancer Res.

[14] Jiang H., Wang X.M. (2009). Highly sensitive detection of daunorubicin based on carbon nanotubes-drug supramolecular interaction. Electrochem. Commun.

[15] Rao G.M., Begleiter A., Lown J.W., Plambeck J.A. (1977). Electrochemical studies of antitumor antibiotics. II. Polarographic and cyclic voltammetric studies of mitomycin C. J. Electrochem. Soc.

[16] Yau H.C., Chan H.L., Yang M. (2003). Electrochemical properties of DNA-intercalating doxorubicin and methylene blue on *n*-hexadecylmercaptan-doped 5′-thiol-labeled DNA-modified gold electrodes. Biosens. Bioelectron.

